# Determinants of Severe Oral Mucositis Development Despite Photobiomodulation Therapy in Stem Cell Transplant Patients

**DOI:** 10.3390/dj13090411

**Published:** 2025-09-08

**Authors:** Sandra Bastos Rezende, Luana Campos, Maria Clara de Souza, Marcos Schoenmann, Maria Cristina Martins de Almeida Macedo, Roberto Luiz da Silva, Alyne Simões

**Affiliations:** 1 German Hospital Oswaldo Cruz, São Paulo 01323-020, SP, Brazil; sandrabrezende@gmail.com; 2 Graduate Program in Dentistry, School of Dentistry, University of Santo Amaro, São Paulo 04829-300, SP, Brazil; lucampos@prof.unisa.br; 3 Undergraduate Program, School of Dentistry, University of São Paulo, São Paulo 05508-000, SP, Brazil; mariaclara1000@usp.br; 4 School of Electronic Engineering, University of São Judas Tadeu, São Paulo 05503-001, SP, Brazil; marcossch@icloud.com; 5 Hematopoietic Stem Cell Transplantation Department, São Camilo Pompeia Hospital, São Paulo 05022-001, SP, Brazil; mariacristina@biosanas.com.br (M.C.M.d.A.M.); roberto@biosanas.com.br (R.L.d.S.); 6 Department of Biomaterial and Oral Biology, School of Dentistry, University of São Paulo, São Paulo 05508-000, SP, Brazil

**Keywords:** Hematopoietic Stem Cell Transplantation, oral mucositis, risk factors, photobiomodulation therapy, dosimetry

## Abstract

**Background/Objectives**: Oral mucositis (OM) is an extremely common and debilitating side effect in patients undergoing Hematopoietic Stem Cell Transplantation (HSCT). As such, managing this condition is an obligatory component for their supportive care. Among the available treatment options, photobiomodulation therapy has long since established itself as the most promising approach, with consistent evidence supporting its efficacy. Despite this, the clinical results of this therapy are still influenced by the chosen dosimetry parameters, as well as patient-specific factors. Therefore, this study aimed to assess which factors can continue to influence the severity and onset of OM despite the protective effect provided by the ongoing photobiomodulation therapy. **Methods**: To achieve this, a retrospective analysis was conducted on the medical records of 171 patients who underwent PBMT during HSCT over a two-year period at the Brazilian Institute of Cancer Control. These files were used to review clinical and laboratorial parameters, such as OM grade, serum CRP, creatinine, urea, OGT, PGT, pain levels, presence of microorganisms in cultures, type of transplant, and conditioning regimens. All of these were evaluated at two different time points: the first day of conditioning and the day of highest OM degree. The statistical analysis was then conducted to evaluate the correlation between the variables and OM grade. **Results**: Results showed that type of conditioning regimens, use of MTX during conditioning, presence of microorganisms in cultures, and increased serum CRP, PGT, and initial urea levels were associated with severe OM. **Conclusions**: Among these, the type of conditioning regimens, MTX usage, positive results in cultures, and increased PGT levels on the first day of conditioning could be considered predictive for severe OM and the worst PBMT results. Consequently, in patients exposed to additional risk factors, adjustments in dosimetry paraments of PBMT or employment of adjuvant therapies should be considered to achieve better results.

## 1. Introduction

Hematopoietic Stem Cell Transplantation (HSCT) is an effective treatment modality for a range of hematological and autoimmune conditions [1]. This multi-step procedure consists of replacing unhealthy bone marrow cells with normal ones sourced from the patient himself (autologous transplant) or a compatible donor (allogeneic transplant) [2]. To enable this, in the days prior to the procedure, high doses of chemotherapy are administered, aiming to reduce the tumor burden by eradicating abnormal cells and enhance conditions for a successful engraftment [3].

Although the conditioning regimen is essential for achieving a positive outcome, it significantly contributes to the occurrence of debilitating side effects [4]. Among these, oral mucositis (OM) is one of the most prevalent [5]. This condition can greatly diminish patients’ quality of life due to its painful symptomatology [6]. The severe pain associated with it, particularly during OM’s ulcerative phase, can impair basic oral functions such as speech, chewing, and swallowing [7]. As a result, patients can experience anorexia and weight loss, as well as weakness, which can, in some cases, lead to the interruption of cancer treatment [8]. Beyond this, OM presence can also prolong the time needed for engraftment and facilitate the emergence of opportunistic infections [9]. Consequently, it not only prolongs hospitalization times and heightens healthcare costs but ultimately increases patients’ morbidity and mortality rates [10]

The significant repercussions of oral mucositis on the primary treatment and its outcomes underscores the importance of addressing this condition [11]. Included in the available interventions for OM are cytoprotective agents, growth factors, systemic or topical analgesics, vitamin E, metabolic supplements, antimicrobials, anti-inflammatory agents, cryotherapy, and gene therapy [12,13]. Many of these treatment modalities, however, play a primarily palliative role, while others are subject to adverse effects or display limited indications or efficacy in the management and prevention of this condition [5,12,14]. Against this backdrop, photobiomodulation therapy (PBMT) has emerged as a valuable preventive and therapeutic strategy since its introduction in the field [15].

This can easily be explained by this therapy’s intrinsic characteristics. PBMT’s success in preventing and halting the progression of OM is attributed to its analgesic, inflammation-modulating, and tissue repair enhancement properties [16,17,18]. As a result, when applied, PBMT provides immediate pain relief while also heightening tissue recovery, something that later translates into a reduced mucositis severity or even its complete prevention. The lack of invasiveness and overall absence of side effects further reinforce its potential as a valuable clinical tool [19,20,21]. Nowadays, PBMT is widely recognized in literature, by both clinical studies and systematic reviews, as one of the mainstays of OM management [22,23,24,25,26,27,28,29]. In HSCT patients who are conditioned with high-dose chemotherapy, the growing body of evidence delving into not only PBMT’s beneficial effects but also its effect on patients’ wellness and prognosis have led to its endorsement by the Multinational Association of Supportive Care in Cancer (MASCC) [12,22,23,24,25,26,27].

Notwithstanding its well-established beneficial effects, PBMT’s outcomes can be significantly influenced by various factors related to the patient, their treatment, and their overall systemic condition [30,31,32,33]. As a result, some patients may deviate from the expected therapeutic trend and continue to experience severe manifestations of OM, even when a standard protocol is used [34,35,36]. Given the existence of a therapeutic window in PBMT and the influence of dosimetry parameters upon it, identifying high-risk patients early on becomes essential to optimize outcomes by tailoring the therapy according to the variables governing individual risk [36,37,38]. Recognizing this, the present study aimed to assess which clinical and laboratory patient parameters could continue to serve as predictive indicators for severe OM, despite the protective effect provided by a standard PBMT protocol, thereby guiding adjustment in treatment choices.

## 2. Materials and Methods

### 2.1. Study Design

In order to initiate the study, ethical approval was obtained from the Research Ethics Committees of the Brazilian Cancer Control Institute (Protocol no. 89774718.7.0000.0075) and the School of Dentistry in University of São Paulo (Protocol no. 89774718.7.3001.0072).

Following approval, a retrospective cross-sectional clinical study was conducted using the medical records of all adult patients who underwent hematopoietic stem cell transplants, either autologous or allogeneic, at the Brazilian Institute of Cancer Control (IBCC), in the time period between April 2017 and March 2019. To be eligible, adhesion to this institution’s general health monitoring protocols, as well as their procedures for prevention and control of OM, was required. On the other hand, the absence of necessary information on patients’ medical forms and the lack of oral mucositis development were considered grounds for exclusion from the study.

Data were collected from the included patients’ medical charts at two different time points: the first day of conditioning and the day with the highest OM severity. The assessed parameters in the physical and electronic files encompassed serum levels of C-reactive protein (CRP), types of pathogenic microorganisms in surveillance cultures or in hemocultures, drug combination used in the conditioning protocols (Table 1, Table S1), reported pain levels, mucositis grades, type of transplant, patients’ gender, occurrence of febrile neutropenia, engraftment times, diagnosis, patients’ death, immunosuppressant usage (Table S1), and patients’ age, as well as the levels of urea, creatinine, oxalacetic glutamic transaminase (OGT), and pyruvic glutamic transaminase (PGT).

### 2.2. Adopted Protocols for Prevention and Control of Oral Mucositis

The hospital’s approach to oral mucositis management in HSCT patients was comprised of three main components: daily mucositis assessment, specialized oral care, and a standardized PBMT protocol.

The PBMT protocols at the hospital consisted of daily irradiations, starting on the first day of transplant conditioning and continuing until the engraftment or the complete remission of all lesions. To achieve this, a Therapy EC (DMC, São Carlos, Brazil) InGaAlP laser device, with a 100 mW power output and a 0.028 cm^2^ spot area was used in conjunction with diode tips. Irradiations were carried out in contact mode, perpendicular to tissue, using continuous waves. In terms of dosimetry parameters, energy was delivered using the 660 nm wavelength to 78 distinct points distributed across the tongue and lining mucosa, with each punctual application delivering 0.2 J over a 2 s period (Figure S1) [11,21,39].

In order to track patients’ evolution and response to applied treatments, the grade of oral mucositis was measured daily using two different scoring criteria. These were defined by the World Health Organization (WHO) and the National Cancer Institute (NCI) [40,41,42,43]. Key parameters evaluated by the grading systems include the condition and integrity of the oral mucosa, as well as patients’ pain levels and oral intake ability (Table S2). The patients’ scores were evaluated every day by two different dental specialists who were also responsible for applying the PBMT. In this study, patients who scored grade 0 in the WHO’s system but had NCI grade 1 were still included.

### 2.3. General Heath Monitoring Protocols Applied for the HSCT Patients

In addition to the strategies for preventing and controlling oral mucositis, all transplant patients were subjected to comprehensive follow-up and monitoring protocols. These encompassed daily pain assessments (before and after PBMT therapy, using a visual assessment scale), regular complementary blood work, and screenings focused on early detection and management of infections. All of these tests aim to closely monitor patients’ conditions and treatment effects, as well as track the occurrence of severe infections, which are major causes of morbidity and mortality in HSCT patients [44].

The hospital’s infection management protocols were established in alignment with international guidelines. These emphasize the importance of regularly sampling patients to facilitate early identification and containment of multidrug-resistant bacteria. This is particularly critical in healthcare settings with high infection rates of carbapenemase-producing Gram-negative bacilli or vancomycin-resistant Enterococcus species. Among the most commonly employed procedures for this purpose are blood and rectal swab surveillance cultures, which enable the detection of microorganisms adhered to the skin and mucosal membranes, even in asymptomatic individuals. This screening is crucial in hindering microbial transmission to other immunocompromised patients who may be more susceptible to severe infections [45,46,47]. Another key guideline is the monitoring of infection biomarkers, such as CRP, through regular blood work. This can be especially useful during neutropenia periods to identify the occurrence of inflammation and severe infection early on, as well as to monitor the results of antibiotic or anti-inflammatory treatments [48].

### 2.4. Statistical Analysis

After collecting and scanning all the data, the IBM SPSS Statistics 24.0 (International Business Machines Corporation, Armonk, NY, USA) Statistics program was used to test the correlation between OM grade (WHO’s scale) and all other independent variables. Initially, the Shapiro–Wilk test was employed to verify the normality of data distribution. Since this test pointed towards a non-normal distribution, non-parametric methods were employed for further analysis. Group comparison tests were performed using the Kruskal–Wallis or the Mann–Whitney U test, depending on the number of groups. Afterwards, Spearman’s rank correlation coefficient was applied to explore the weight of each variable over the OM grade. A significance level of 5% was considered for all the statistical tests.

## 3. Results

### 3.1. Study Population

During the data collection phase in this study, a total of 171 adult patients had their medical charts reviewed by convenience. However, only 141 were deemed eligible (Table 1), since six charts (3.5%) lacked the necessary information, and 24 patients (14%) did not develop any level of OM, scoring grade 0 across all OM assessment scales.

**Table 1:** Summary of patient demographic characteristics, including gender distribution, age range, mean oral mucositis scores (WHO scale), and type of conditioning regimens, according to hematopoietic stem cell transplant modality.

Gender	n	Percentage	OM Mean *	Age Range (Years)
Male	71	50.35	2.23	17–75
Female	70	49.65	2.51	21–72
Conditioning Regimens **	Allogeneic transplants		Autologous transplants	
	n	Percentage	n	Percentage
BEAC	1	0.71	7	4.96
BEAM	0	0	5	3.55
Bu-Cy	12	8.51	1	0.71
Bu-Flu	26	18.44	0	0
BuMel	7	4.96	2	1.42
Cy-TBI	14	9.93	0	0
Flu-Bu-Cy	18	12.77	0	0
Flu-Cy	3	2.13	0	0
Flu-Cy-TBI	11	7.8	0	0
Flu-Melphalan	9	6.38	0	0
Melphalan	0	0	25	17.73
Total	101	71.63	40	28.37

* Average of the maximum oral mucositis (OM) scores recorded per patient, measured using the World Health Organization (WHO) grading scale. ** Details regarding the conditioning regimens can be found in Table S1 and in [49]. n = number of patients. OM = oral mucositis. BEAC = carmustine, etoposide, cytarabine, and cyclophosphamide. BEAM = carmustine, etoposide, cytarabine, and melphalan. Bu = busulfan. Cy = cyclophosphamide. Flu = fludarabine. Mel = melphalan. TBI = total body irradiation.

### 3.2. Correlation Between the Independent Variables and OM Grade

#### 3.2.1. Treatment Parameters

**Figure 1:**
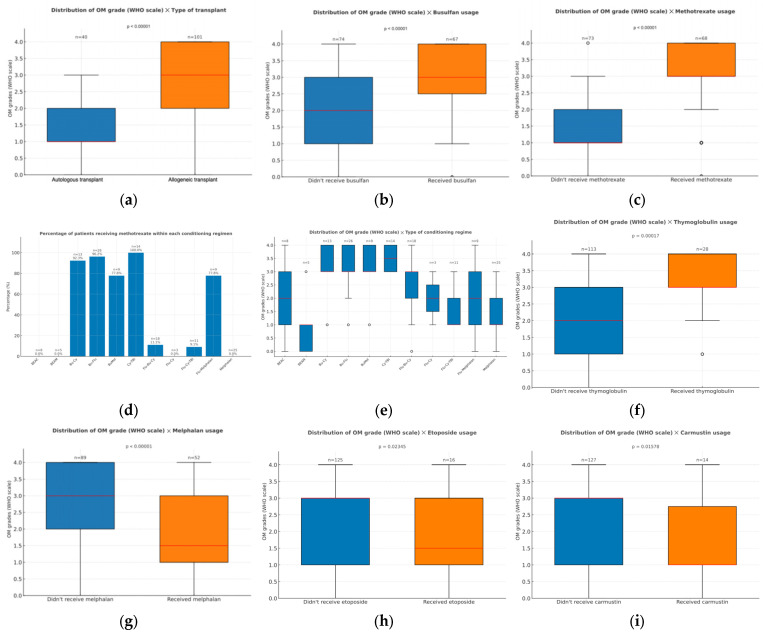
(**a**) Distribution of oral mucositis grades (WHO scale) according to the transplant type (autologous or allogeneic); (**b**) Distribution of oral mucositis grade (WHO scale) according to the usage of busulfan during conditioning; (**c**) distribution of oral mucositis grade (WHO scale) according to the usage of methotrexate during conditioning; (**d**) percentage of patients receiving methotrexate within each conditioning regimen; (**e**) distribution of oral mucositis grade (WHO scale) according to the conditioning regimen employed for the transplant; (**f**) distribution of oral mucositis grade (WHO scale) according to the usage of thymoglobulin during graft-versus-host-disease prophylaxis; (**g**) distribution of oral mucositis grade (WHO scale) according to the usage of melphalan during conditioning; (**h**) distribution of oral mucositis grade (WHO scale) according to the usage of etoposide during conditioning; (**i**) distribution of oral mucositis grade (WHO scale) according to the usage of carmustin during conditioning. Outliers are displayed as circles, defined as values greater than 1.5 times the interquartile range (IQR) above the upper quartile or below the lower quartile.

In this study, individuals who underwent allogeneic transplants demonstrated higher OM severity (*p* < 0.001). Their WHO scores showed higher medians (around grade 3) and a wider range (grades 0 to 4), whereas autologous transplant recipients exhibited a lower median score (around grade 1), with no cases exceeding grade 3 (Figure 1a).

Another important factor influencing mucositis development was the types of medications used for conditioning and graft-versus-host-disease (GVHD) prophylaxis. Busulfan (*p* < 0.001) usage during the conditioning phase was associated with a higher severity of oral mucositis (Figure 1b). Among patients who developed the highest OM grade, approximately 70% had received busulfan, whereas this proportion dropped to about 10% in those with WHO grade 0. This medication, however, was often co-administrated with methotrexate (MTX), which was also linked to higher incidence and severity of oral mucositis (*p* < 0.001; Figure 1c). Notably, over 95% of patients who developed OM grade 4 had been treated with MTX, whereas fewer than 10% of those with grade 0 had been exposed to this immunosuppressant. Reflecting this, the conditioning regimens that showed a higher severity of OM were also the ones that used MTX, such as Bu-Cy, Bu-Flu, BuMel, and Cy-TBI (Figure 1d,e). In addition, thymoglobulin (*p* < 0.001) usage in the GVHD prophylaxis was also associated with severe mucositis, since higher grades of OM were observed in the 28 patients who received this medication (Figure 1f). On the other hand, patients treated with melphalan (*p* < 0.001), etoposide (*p* = 0.024), or carmustine (*p* = 0.016) presented slightly lower OM grades, compared to those who received other conditioning regimens without these agents (Figure 1g–i). None of the other medications employed (including cyclophosphamide, cytarabine, fludarabine, thiotepa, and mesna) showed a significant correlation to OM severity (*p* > 0.05). Total body irradiation also did not present any significant correlation with OM.

#### 3.2.2. Presence of Microorganisms in Cultures

**Figure 2:**
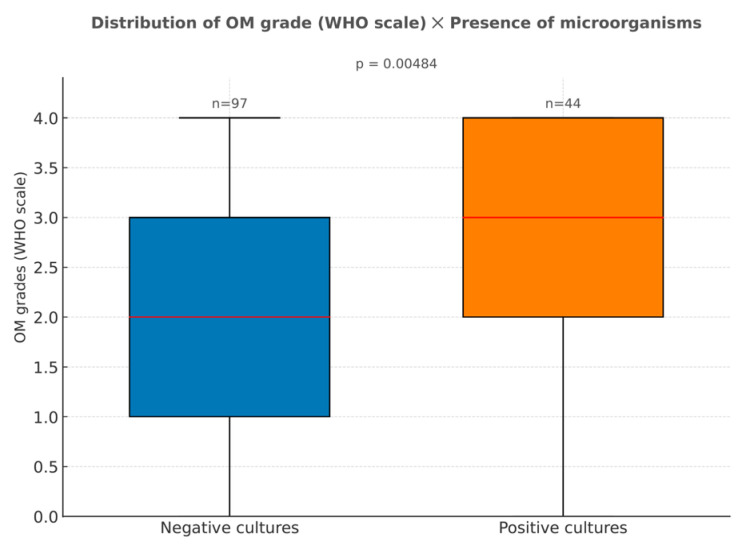
Distribution of oral mucositis grades (WHO scale) according to the presence of microorganisms in cultures. Negative results in the hemocultures or swabs cultures showed the absence of microorganisms. Positive results in either type of surveillance culture showed the presence of microorganism.

The presence of microorganisms in cultures was associated with the development of higher grades of oral mucositis (*p* = 0.005; Figure 2). In the patients with positive hemocultures, the most common types of microorganisms found were Cytomegalovirus, *Staphylococcus epidermis*, *Stenotrophomonas maltophilia*, and *Escherichia coli*. Meanwhile, the rectal swabs cultures showed higher prevalences of *Escherichia coli*, *Klebsiella pneumoniae*, *Enterobacter cloacae complex*, and *Enterococcus faecium*.

#### 3.2.3. Laboratorial Parameters

**Figure 3:**
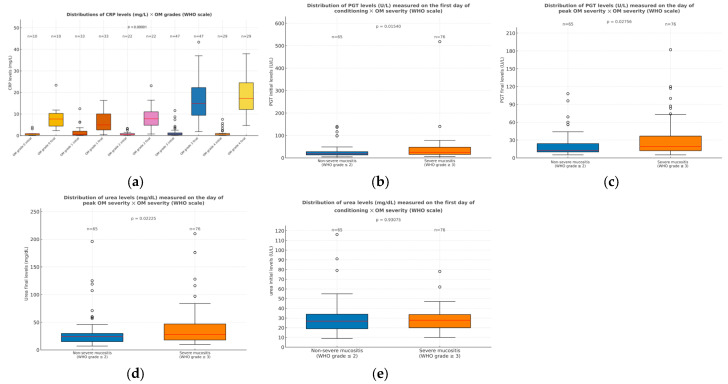
(**a**) Distributions of CRP levels (mg/dl) across OM grades at two different time points: the first day of conditioning and the day of peak OM severity. Significant differences between oral mucositis grades were only observed for CRP levels on the day of highest OM (*p* < 0.001). At this time point, CRP levels were significantly higher in patients with severe mucositis (grades 3 and 4), compared to those with mild or no mucositis (grades 0, 1, and 2), with adjusted *p*-values indicating statistically significant differences, particularly between grades 0 and 4 (*p* = 0.017), 1 and 3 (*p* < 0.001), 1 and 4 (*p* < 0.001), 2 and 3 (*p* = 0.007), and 2 and 4 (*p* < 0.001). No significant differences were found between other grade comparisons. (**b**) Distribution of PGT levels measured on the first day of conditioning in patients with non-severe (grades 0 to 2) and severe mucositis (grades 3 and 4); (**c**) distribution of PGT levels measured on the day of peak OM in patients with non-severe (grades 0 to 2) and severe mucositis (grades 3 and 4); (**d**) distribution of urea levels measured on the day of peak OM in patients with non-severe (grades 0 to 2) and severe mucositis (grades 3 and 4); (**e**) distribution of urea levels measured on the first day of conditioning in patients with non-severe (grades 0 to 2) and severe mucositis (grades 3 and 4). No significant differences were observed at this time point. Outliers are displayed as circles, defined as values greater than 1.5 times the interquartile range (IQR) above the upper quartile or below the lower quartile.

CRP levels measured on the day oral mucositis reached its peak also showed a reciprocal correlation with the mucositis grade (*p* < 0.001). For individuals with low-grade mucositis, median CRP values on the day of the highest OM score were approximately around 7 mg/L. In contrast, those with severe OM (grades 3 and 4) exhibited median CRP levels around 17 mg/L, ranging up to 44 mg/L in some patients. The values for both groups were above the normal reference range, but in severe mucositis, the levels were markedly elevated, resembling those seen in severe infections. Interestingly, no correlation was observed between the initial CRP values (collected on the first day of conditioning) and the OM grade (*p* > 0.05), even if a progressive increase was observed along the worsening of the patient’s oral conditioning (Figure 3a).

Patients with higher levels of pyruvic glutamic transaminase on the first day of conditioning also progressed to direr clinical manifestations of OM. Specifically, those who later presented severe mucositis (grades 3 and 4) exhibited higher PGT levels on the first day of conditioning (*p* = 0.016), even if these values still remained within the normal reference range at that point (Figure 3b). Furthermore, PGT levels on the day of maximum mucositis severity were also higher among patients who exhibited more intense clinical manifestations (grades 3 and 4) than in those with milder symptoms (*p* = 0.027; Figure 3c).

Severe oral mucositis was additionally correlated with serum urea levels, in the sense that those with worse clinical conditions exhibited higher blood concentrations on the day mucositis was at its peak (*p* = 0.023; Figure 3d). However, elevated urea levels at the start of conditioning did not predict the subsequent severity of mucositis, since no significant differences were found in urea levels on the first day of conditioning across mucositis grades (*p* > 0.05; Figure 3e).

Lastly, neither creatinine nor OGT serum concentrations, at conditioning onset or during the OM peak, were significantly associated with mucositis severity (*p* > 0.05).

#### 3.2.4. Engraftment Times

**Figure 4:**
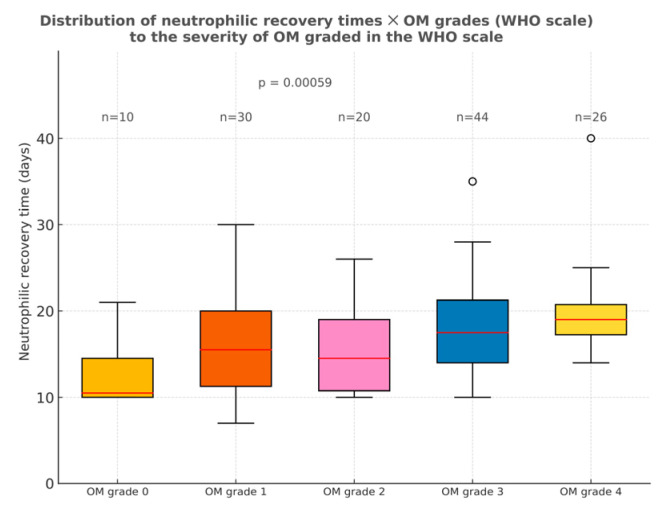
Distribution of neutrophilic recovery times according to the severity of OM graded in the WHO scale. Comparison of neutrophilic recovery times and the maximum OM grade was performed using the Kruskal–Wallis and Dunn’s tests. In these, significant differences were found between individuals with OM grade 0 and those with grades 3 (*p* = 0.014) and 4 (*p* = 0.004), as well as between grade 2 and grade 4 (*p* = 0.037). Outliers are displayed as circles, defined as values greater than 1.5 times the interquartile range (IQR) above the upper quartile or below the lower quartile.

Furthermore, the statistical analysis revealed a correlation between delayed engraftment times and the severity of oral mucositis (*p* < 0.001). In patients with OM grade 0, engraftment occurred around the 10th day post-transplant. This interval progressively lengthened with the increase in oral mucositis grade, nearly doubling in those with grade 4 mucositis (Figure 4). As a result, statistically significant differences in engraftment times were observed when comparing grade 0 to grades 3 (*p* = 0.014) and 4 (*p* = 0.004) and between grades 2 and 4 (*p* = 0.037). These findings reinforce the hypothesis that higher severity of mucositis may play a significant role in delaying hematopoietic recovery.

#### 3.2.5. Pain Levels When Oral Mucositis Was at Its Peak

**Figure 5:**
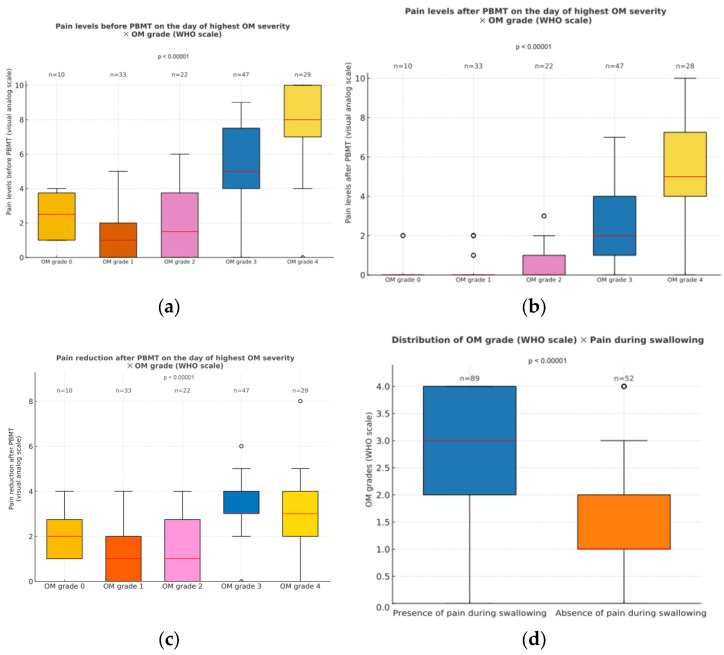
(**a**) Pain levels before PBMT on the day of highest OM severity, recorded through a visual analog scale, across the different OM grades (WHO scale). Significant differences were observed between grades 0 and 3, 0 and 4, 1 and 3, 1 and 4, 2 and 3, and 2 and 4, as well as grades 3 and 4 (*p* < 0.05); (**b**) Pain levels at the height of OM severity, after PBMT, across the different OM grades (WHO scale). Significant differences were seen between grades 0 and 3, 0 and 4, 1 and 3, 1 and 4, 2 and 3, and 2 and 4, as well as grades 3 and 4 (*p* < 0.05); (**c**) change in pain levels (pre-PBMT minus post-PBMT) recorded on the day of highest OM severity, shown by OM grade (WHO scale). Significant differences were observed between the following grades: 0 and 3, 1 and 3, 1 and 4, 2 and 3, and 2 and 4; (**d**) distribution of OM grades according to the presence or absence of pain during swallowing. Outliers are displayed as circles, defined as values greater than 1.5 times the interquartile range (IQR) above the upper quartile or below the lower quartile.

The overall pain levels reported on the day of highest mucositis degree were associated with mucositis intensity (Figure 5). Patients displaying severe mucositis experienced higher general pain levels (*p* < 0.001), with some individuals reporting maximum values on the pain scale (Figure 5a). Interestingly, these patients were also the ones who demonstrated the greatest absolute reduction in pain following PBMT (*p* < 0.001; Figure 5b,c), even if their pain intensity remained higher than in those with milder conditions (*p* < 0.001). This trend was evident in the comparison of pain medians before and after PBMT across different severity groups: before PBMT, patients with grade 4 mucositis reported median pain levels around 8, while those with grades 0 to 2 reported values near 2 (Figure 5a). After PBMT, pain in the lower-grade group dropped to nearly 0, whereas individuals with grade 4 still exhibited median levels close to 5 (Figure 5b,c). Swallowing-related pain was also more common in patients with higher OM grades (*p* < 0.001; Figure 5d).

## 4. Discussion

Photobiomodulation therapy (PBMT) has proven, over the years, to significantly enhance both the quality of life and overall survival of patients undergoing HSCT [8,22]. Its effectiveness in promoting tissue repair and alleviating pain has contributed to a reduction in complications and healthcare costs, establishing its value as a key component of their supportive care [11,17]. In our study, a relationship between oral mucositis and reported pain levels, as well as engraftment times, was observed. As such, PBMT’s proven ability in reducing OM burden [15,24] could indirectly lead to shorter engraftment times and decreased pain levels. Both of these outcomes not only play a role in facilitating oral intake and reducing the risk of local and systemic infections but also contribute to minimizing the length of hospital stays and the likelihood of treatment interruption due to orofacial complications [6,8,14,22]. Consequently, our findings may lend further support to the notion that PBMT is capable of aiding the overall therapeutic process and improving patient prognosis.

Despite widespread recognition of its benefits, ongoing research still seeks a better understanding about the factors influencing PBMT’s outcomes, with the goal of refining and individualizing treatment protocols for more consistent and personalized results [31,50,51,52]. In this study, a series of patient-related factors that can shape mucositis progression despite the positive impact of PBMT was examined. Amidst the independent variables found to have a significant correlation to OM development, we can name the type of transplant, medications used for the conditioning and GVHD prophylaxis, and presence of microorganisms in cultures, as well as the serum levels of CRP, urea, and pyruvic glutamic transaminase.

Regarding the type of transplant, severe clinical stages of OM were more frequently observed in allogeneic transplants when compared to autologous transplants. The chemotherapy agents employed during conditioning oftentimes differ according to the donor source of the transplant. Although in our article, some conditioning regimens were employed for both types of transplants, this is an exception motivated by clinical decisions due to the patients’ disease type, age, or other comorbidities [49,53]. Additionally, recipients of allogeneic hematopoietic transplants undergo not only a heavier conditioning phase (with full-body radiation and specific chemotherapy agents that are more stomatotoxic) but also receive immunosuppressive agents post-transplant, aiming to prevent graft-versus-host disease [54,55,56]. In turn, these patients often experience longer-lasting neutropenia, a fact that increases their susceptibility to bacterial and fungal infections post-transplant [57,58]. The higher the frequency of post-transplant bacterial or fungal infections, the longer the engraftment times, and the intrinsic actions of chemotherapy agents used during conditioning in these types of transplants ultimately favor the occurrence of higher forms of OM [54,59].

Chemotherapeutic agents used during conditioning were also shown to influence OM severity. Specifically, MTX was significantly associated with more frequent, prolonged, and severe cases of mucositis [60]. This can be attributed to MTX’s pharmacological action, wherein it disrupts DNA synthesis by mimicking folic acid molecules and inhibiting its normal metabolic pathway. Such a mechanism is highly associated with a stomatotoxic effect, as it disrupts cell proliferation, hindering repair and ultimately aggravating OM’s presentation [61]. This is supported by various studies that link more pronounced mucositis presentation to MTX, particularly when compared to either the absence of MTX or its use at lower dosages [34,62].

Worsened PBMT results were also observed when busulfan or thymoglobulin was employed. The former, an alkylating agent widely employed in conditioning protocols, is known for its cytotoxic effects on the nervous system, liver, and mucosal tissues [63]. In light of this profile, it is unsurprising that busulfan could contribute to the onset of severe mucositis in a dose-dependent manner [64]. In most of the patients in this study, however, busulfan was not administrated in isolation but rather in association with methotrexate and other chemotherapy agents. In fact, out of the 67 patients who received busulfan, 46 also received MTX, while the remaining ones were also subjected to other pharmacological agents. Owing to this, isolating the individual impact of each drug over PBMT effectiveness is not as straightforward, since the observed results for busulfan reflect the cumulative impact of both agents. A similar scenario can be observed for thymoglobulin administration. Although this immunosuppressive agent is not classically considered stomatotoxic [65,66], its administration is generally reserved for patients that are already at a greater risk, most notably those undergoing allogeneic transplants and who are therefore at risk of GVHD, who also receive MTX as part of their immunosuppressive regimen. In our study, 92.86% of those who received thymoglobulin were also administrated MTX, further confirming that the observed impact on OM may be attributed to factors beyond the use of thymoglobulin alone.

Meanwhile, melphalan, carmustin, and etoposide were associated with milder cases of mucositis and better PBMT results. The observed lowered toxicity, however, may be partially attributed to the fact that all three medications are more commonly employed in conditioning for autologous transplants, which generally show more favorable outcomes regarding OM development. Given this context, in these patients, employing PBMT along with cryotherapy may often be sufficient to prevent the onset of severe OM [67,68,69].

As for the presence of microorganisms in surveillance cultures, it can be regarded both as a contributing factor and a byproduct of OM [70,71]. This dual role arises because a superimposed infection can exacerbate the tissue damage and amplify the inflammation seen in the pathobiological phases of OM development and progression [71]. Simultaneously, the loss of mucosal integrity and, as a result, of its barrier function serves as a risk factor for systemic infections in neutropenic patients. Hence, the oral cavity, along with the entire gastrointestinal tract, acts as a major gateway for infections in transplant recipients [72,73]. Given this, it is no surprise that a correlation exists between the presence of pathogenic microorganisms in surveillance cultures and OM grade, as well as longer engraftment times and transplant complications.

Furthermore, an observable relationship emerged between OM and CRP concentration in the bloodstream. This can mostly be explained by the fact that CRP is an acute phase protein that acts as an inflammatory marker [74]. As such, the production of this protein is fostered by the synergic action of proinflammatory cytokines, such as interleukin-1, tumor necrosis factor alpha, and specially interleukin-6 [75]. The production of these factors can, in turn, be sustained by the inflammation occurring in OM, as well as its endorsement of the microorganism’s translocation and entry into the tissue or circulation. Owing to this, CRP levels can be elevated during mucositis, often peaking alongside the most pronounced stages of OM or even in the presence of secondary infection [76,77,78].

Beyond CRP, serum levels of PGT and urea were also evaluated in relation to OM severity. Clinically, the first is a hepatic enzyme most commonly used to assess liver damage, alongside OGT, alkaline phosphatase, and gamma-glutamyl transferase. Meanwhile, the second is often used together with creatinine levels as part of a standard kidney function panel. In literature, the impact of HSCT over renal or hepatic function was explored, with some studies establishing a relationship between the transplant itself and subsequent organ dysfunction, especially in the long term, either due to conditioning toxicity, secondary infections, or GVHD complications [79,80,81,82,83]. Nevertheless, the correlation between oral mucositis and impaired liver or hepatic function is still unclear. One particular study observed an association between OM grade and hepatic and renal markers, suggesting that mucositis severity could worsen when organ function is compromised, possibly due to delayed excretion of chemotherapeutic agents or other drugs used during treatment [35]. This hypothesis is further supported by a few other studies that correlated delayed MTX clearance and increased incidence of severe OM [84] or even observed that serum creatinine levels can be used to monitor plasma MTX concentrations [85]. Our particular study identified an association between OM grade and some hepatic and renal biomarkers, since significant differences in PGT and final urea levels were observed, despite the absence of meaningful changes in other parameters like OGT and creatinine. Thus, our findings may lend further support to the potential link between renal or hepatic dysfunction and OM severity, even if further investigation is warranted to clarify this relationship. If future studies can determine more precise threshold values for these biomarkers and solidify the relationship between OM severity and renal or hepatic function, they could prove useful for early risk stratification of patients more likely to develop severe OM.

All of the aforementioned factors that function as predictors for OM severity can serve as a basis for recommending individualized treatment strategies. Protocol individualization based on risk stratification is essential, as it may not be feasible to provide fully individualized treatment for all patients due to hospitals’ logistical constraints, such as the time and staff availability that would be required for it. Furthermore, individualizing the protocol may not be necessary for patients who already achieve satisfactory outcomes with the standard treatment. Hence, adjusting PBMT parameters or even combining it with other strategies could be recommended when factors that indicate a higher risk of suboptimal OM outcomes despite the application of a standard PBMT protocol are at play [36].

Among the parameters that could be considered for adjustment are irradiance, potency, time per point, dose, energy, frequency of applications, number of points, and wavelength [86,87]. Various previous studies have begun exploring the impact of these variables on PBMT-induced repair, with some indicating that lower energy densities might elicit better healing responses in the oral mucosa than high ones [30,88]. As such, in higher-risk patients, for instance those receiving MTX, performing irradiations more frequently, such as twice daily, could prove more beneficial than increasing the energy delivered per area. Combining PBMT with adjuvant interventions, notably photodynamic therapy and cryotherapy, should also be explored, since the former has already showed a capability to improve OM by reducing the microbial load, while the latter is effective in diminishing the damage caused in the oral cavity during chemotherapy infusion [67,89,90]. Such associations may prove particularly beneficial for patients harboring positive microbial cultures or those receiving medications known for their heightened stomatotoxicity. These suggestions, however, warrant further support by comprehensive clinical studies, as the combined influence of PBMT’s various dosimetric factors and potential associations with other therapies, necessary to establish effective guidelines, remain underexplored.

## 5. Conclusions

Overall, the statistical analysis revealed that the following factors could be considered predictive for severe oral mucositis during HSCT: the type of transplant; the conditioning regimens, especially those using MTX; the presence of microorganisms in cultures; and increased serum PGT levels on the first day of conditioning. Consequently, tailoring PBMT’s parameters and using complementary management strategies may be necessary for patients exposed to these factors.

## Data Availability

The data presented in this study are stored in the author’s institutional drive and are not publicly available due to privacy and ethical restrictions, as they involve patient information. The raw data supporting the results and conclusions of this article will be made available by the authors upon request.

## Supplementary Materials

The following supporting information can be downloaded at: https://www.mdpi.com/article/10.3390/dj13090411/s1, Figure S1: Depiction of the applied PBMT protocol; Table S1: Types of drugs used in the conditioning regimens or for the prophylaxis and management of graft-versus-host disease in the patients undergoing HSCT; Table S2: Grading systems for oral mucositis.

## Abbreviations

The following abbreviations are used in this manuscript:

OM	Oral mucositis
HSCT	Hematopoietic Stem Cell Transplantation
PBMT	Photobiomodulation therapy
MTX	Methotrexate
CRP	C-reactive protein
OGT	Oxalacetic glutamic transaminase
PGT	Pyruvic glutamic transaminase
MASCC	Multinational Association of Supportive Care in Cancer
WHO	World Health Organization
NCI	National Cancer Institute
IBM SPSS	International Business Machines Corporation
BEAC	Carmustine, etoposide, cytarabine, and cyclophosphamide
BEAM	Carmustine, etoposide, cytarabine, and melphalan
Bu	Busulfan
Cy	Cyclophosphamide
Flu	Fludarabine
Mel	Melphalan
TBI	Total body irradiation
GVHD	Graft-versus-host disease
