# Supplementary material for: Determinants of Severe Oral Mucositis Development Despite Photobiomodulation Therapy in Stem Cell Transplant Patients
Source: Dent J (Basel). 2025 Sep 8;13(9):411. doi: 10.3390/dj13090411 (PMC12468854; doi:10.3390/dj13090411)
Supplement: Supplementary file 1 [file dentistry-13-00411-s001.zip › dentistry-3760906-Supplementary materials.pdf]

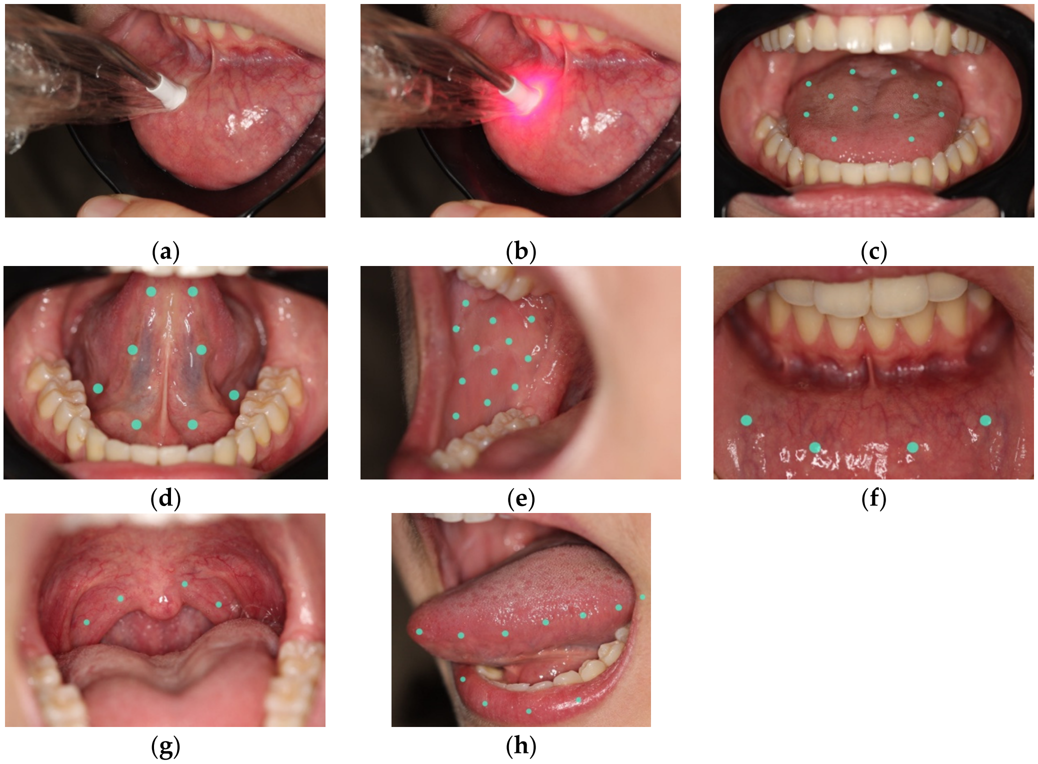

Supplementary Figure S1. Depiction of the applied PBMT protocol. (**a-b**) Representation of PBMT's application method, keeping the device in contact mode and perpendicular to the targeted tissue. (**c-h**) Representation of PBMT's irradiation sites across the lining mucosa, distributed as follows: 12 on the dorsum of the tongue (6 on each side), 4 on the floor of the mouth (2 on each side), 4 on the ventral surface of the tongue (2 on each side), 12 on each buccal mucosa, 8 on the labial mucosa (4 on the upper and 4 on the lower), 4 on the soft palate (2 per side), 6 on each of the lateral borders of the tongue, 8 on the lip vermilion (4 upper and 4 lower), and 2 on the oral commissures (1 on each side)

Supplementary Table S1. Types of drugs used during the conditioning regimens or for the management of graft-versus-host disease in the patients undergoing HCT.

**Conditioning regimens**[1]	**Dose**	**Frequency**
<u>BEAM</u>		
Carmustine (mg/m²)	300-400	D-6
Etoposide (mg/m²)	100-200	D-5 to D-2 (12/12h)
Cytarabine (mg/m²)	200-400	D-5 to D-2 (12/12h)
Melphalan (mg/m²)	140	D-1
<u>BEAC</u>	300-400	D-6
Carmustine (mg/m²)	100-200	D-5 to D-2(12/12h)
Etoposide (mg/m²)	100-400	D-5 to D-2(12/12h)
Cytarabine (mg/m²)	35	D-5 to D-2
Cyclophosphamide (mg/kg/day)		
<u>BuMel</u>		
Busulfan (mg/m²)	130	D-7 to D-4
Melphalan (mg/m²)	70	D-2 to D-1
<u>Melphalan</u>	200	D-1
Melphalan (mg/m²) **<u>or</u>**	140	D-1
Melphalan (mg/m²) **<u>or</u>**	800	D-1
Melphalan (mg/m²) with TBI (cGy)		

Bu-Cy<br>Busulfan (mg/kg/day)<br>Cyclophosphamide (mg/kg/day)	3.2<br>50	D-7 to D-4<br>D-3 and D-2
Bu-Flu[2]<br>Busulfan (mg/kg/day)<br>Fludarabine(mg/m²/day) **or**<br>Fludarabine (mg/m²/day)	 3.2<br>40<br>30	 D-6 to D-3<br>D-6 to D-3<br>D-6 to D-2
Cy-TBI<br>Cyclophosphamide (mg/kg/day)[3]<br>Fractionated TBI (Gy)[4]	60<br>10-12	D-6 and D-5<br>D-3, D-2 and D-1
Flu-Cy-TBI with Cy post-transplant<br>Cyclophosphamide (mg/kg)<br>Fludarabine (mg/m²)<br>TBI (cGy)<br>Cyclophosphamide (mg/kg)	14,5<br>30<br>200<br>50	D-6 and D-5<br>D-6 to D-2<br>D-1<br>D+3 and D+4
Flu-Bu-Cy with Cy post-transplant<br>Fludarabine (mg/m²)<br>Busulfan (mg/m²)<br>Cyclophosphamide (mg/kg)<br>Cyclophosphamide (mg/kg)	 25<br>110-130<br>14.5<br>50	 D-6 to D-2<br>D-7 to D-4<br>D-3 and D-2<br>D+3 and D+4
Flu-Cy<br>Fludarabine (mg/m²/day)<br>Cyclophosphamide (mg/kg/day)	25<br>60	for 5 days<br>for 2 days
Flu-Melphalan<br>Fludarabine (mg/m²/day) **or**<br>Fludarabine (mg/m²/day) **or**<br>Fludarabine (mg/m²/day)<br>Melphalan (mg/m²)	 25<br>30<br>30<br>140	 for 5 days<br>for 5 days<br>for 4 days<br>for 1 day
**Immunosuppressive drugs**	**Dose**	**Frequency**
Cyclosporine (mg/L)	200-300<br>100-200	Starts at D-1<br>after 3 or 4 weeks
Tacrolimus (mg/kg)	0.02	Starts at D-3
Methotrexate (MTX) (mg/m2)	5-10	D+1,D+3,D+6,D+11
Mycophenolate Mofetil (mg/kg)	30-45	D+5 to D+35
Cyclophosphamide (mg/kg)	50	D+3 and D+4

[1] The names of the conditioning regimens are standardized within the field and refer to the medications used in each regimen, as detailed in the table.
[2] This conditioning protocol may vary
[3] Days of administration may vary
[4] TBI doses and days may vary according to disease status

Supplementary Table S2. Grading systems for oral mucositis.

	WHO	NCI	
		Clinical exam	Symptoms/Functional exam
Grade 0	No mucositis	No mucositis	No mucositis
Grade 1	Erythema and soreness	Erythema	Asymptomatic or mild symptoms, patients retain a normal/solid diet
Grade 2	Ulcers, still able to eat solids	Ulcers or pseudomembranes	Moderate pain, patients require a modified diet

Grade 3	Confluent ulcers, liquid diet only	Confluent ulcers or pseudomembranes with trauma that can lead to bleeding	Severe pain, oral intake is not possible
Grade 4	Confluent ulcers, presence of bleeding, oral intake is not possible	Tissue necrosis, spontaneous bleeding and life-threatening consequences	Symptoms associated with life threatening risk

WHO = World Health Organization. NCI = National Cancer Institute.
